# Multilayered Gel-Spotting Device for *In Vitro* Reconstruction of Hair Follicle-like Microstructure

**DOI:** 10.3390/mi14091651

**Published:** 2023-08-22

**Authors:** Aki Sugeno, Takahiro Sumi, Hanako Sato-Yazawa, Takuya Yazawa, Hajime Inoue, Shogo Miyata

**Affiliations:** 1 Graduate School of Science and Technology, Keio University, 3-14-1 Hiyoshi, Yokohama 223-8522, Japan; 2 Department of Pathology, Dokkyo Medical University, Tochigi 321-0293, Japan; hnyazawa@dokkyomed.ac.jp (H.S.-Y.); tkyazawa@dokkyomed.ac.jp (T.Y.); 3 Department of Plastic and Reconstructive Surgery, St. Marianna University School of Medicine, Kawasaki 216-8511, Japan; h2inoue@marianna-u.ac.jp; 4 Department of Mechanical Engineering, Faculty of Science and Technology, Keio University, 3-14-1 Hiyoshi, Yokohama 223-8522, Japan

**Keywords:** hair follicle, *in vitro* regeneration, cytokine concentration gradient, multilayered gel culture, mouse embryonic stem cell, epidermal cell

## Abstract

Hair follicles play an important role in hair development. This study aimed to develop a microgel-spotting device to fabricate a multilayered gel bead culture model and to mimic the early development of skin appendages to regenerate hair follicles *in vitro*. The model consists of an alginate gel layer containing cytokines as the core layer, a collagen gel layer containing mouse embryonic stem cells as the middle layer, and a collagen gel layer containing fetus-derived epidermal cells as the outer layer. A concentration gradient of cytokines is formed, which promotes interactions between epidermal and stem cells. Histological and immunnohistological analyses confirmed the reconstruction of hair follicle structures. As a result, the cell number and gel bead size could be precisely controlled by the developed microgel-spotting device. In the multilayered gel bead, the embryonic and epidermal cells cultured with the cytokine gradient formed cell aggregates with keratinized tissue in the center similar to “native” hair follicle structure. Sweat gland-like luminal tissue and erector pilorum-like structures were also observed around aggregates with concentric structures. In conclusion, the multilayered gel bead culture model demonstrated potential for *in vitro* hair follicle regeneration. The findings of this study provide insight into the early development of skin appendages.

## 1. Introduction

Hair follicles are formed during the fetal period through interactions between embryonic skin epithelial cells in the epidermis and mesenchymal stem cells in the dermis. During hair follicle development, the layer of fetal epidermal cells thickens to form a placode, and dermal cells aggregate under the placode. Hair follicles can be regenerated by mimicking this developmental process [1]. However, the mechanisms that trigger hair follicle development are not fully understood.

Various three-dimensional (3D) culture methods have been developed to enhance the *in vitro* hair follicle regeneration. *In vitro* 3D culture requires mimicking *in vivo* structures and interactions between skin epithelial and mesenchymal cells [2,3]. Previously, dermal papilla (DP) cells, keratinocytes, and outer root sheath (ORS) cells have been co-cultured two-dimensionally or three-dimensionally in collagen gel or Matrigel, or a sandwiched culture of these gels [4,5,6,7]. Co-culture of keratinocytes and ORS cells from human hair follicles encapsulated in collagen gel or Matrigel have revealed epidermoid cyst-like spheroids and spike-like structures [7]. Mixed cell aggregates of embryonic mouse follicular dermal and epidermal cells formed using the hanging drop method can generate protohair structures [8]. These approaches are superior for studying the development of hair follicles; however, they limit the possibilities of clinical application because they require cells derived from hair follicles. Kageyama et al. reported that spheroids self-organize to form dumbbell-shaped conjugate using a mixed culture of embryonic mouse skin epithelial and mesenchymal cells [9]. Hair follicles regenerated after grafting the cell conjugates onto the back skin of nude mice. However, *in vitro* regeneration of hair follicles and hair appendages has not been completely performed. 

In this study, we established a multilayered gel bead culture model to simulate the early development of skin appendages. Mouse embryonic stem cells (mESCs) and fetal epidermal cells were cultured in this multilayered culture to promote cell–cell interactions. A cytokine concentration gradient was generated using the culture model. The multilayered culture was analyzed by immunnohistological staining of specific hair follicle components. 

## 2. Materials and Methods

### 2.1. Experimental Design for In Vitro Reconstruction of Skin Appendages

**Figure 1:**
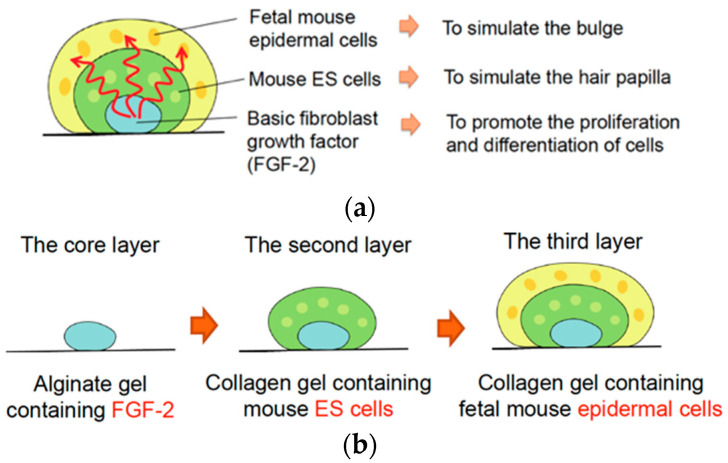
Schematic representation of (**a**) three-layered microgel spot culture for skin appendage regeneration and (**b**) fabrication process of the layered gel construct.

In this study, we proposed a three-dimensional culture method to simulate the skin tissue structure in an early development stage. This method used a gel microconstruct consisting of an alginate gel layer containing cytokines as the core layer, a collagen gel layer containing pluripotent stem cells as the middle layer, and a collagen gel layer containing fetus-derived epidermal cells as the outer layer (Figure 1). FGF-2, which promotes skin cell differentiation and maturation [10], is contained in the core layer, mouse embryonic stem cells (ESCs) in the middle layer, and fetus-derived mouse epidermal cells in the outer layer. A cytokine concentration gradient generated toward the outer layer promoted the interaction between epidermal and ES cells to regenerate multilayered skin structures, including skin appendages. 

### 2.2. Precise Microgel-Spotting Device for Fabricating Multilayered Gel Constructs Containing Cells

**Figure 2:**
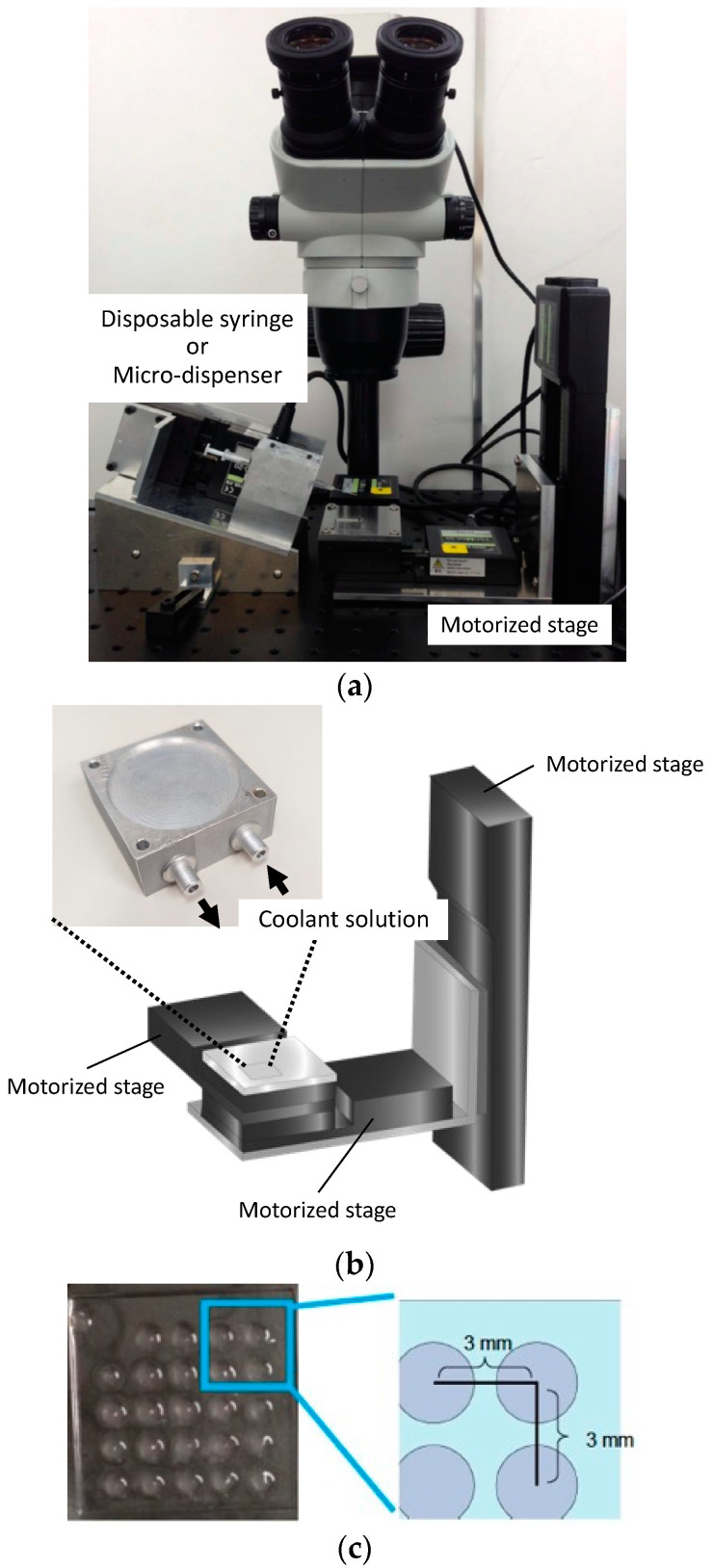
(**a**) Mechanical spotting system for cell-suspended collagen solution that fabricated multilayered gel constructs. (**b**) The position of spotting point was controlled using three motor-driven stages. (**c**) Gel constructs were fabricated with precisely positioned array.

A precisely controlled gel solution-spotting device was constructed to array cell-containing microgel constructs. The device consisted of a custom-made microinjector for cell-containing collagen or cytokine-containing alginate solutions and a spotting stage with three-axis position control (Figure 2a).

The microinjector consisted of a motorized stage (TSDM60-20X, Opto-Sigma, Japan) and a 1 mL disposable syringe (SS-01T, TERUMO, Tokyo, Japan) or microdispenser (5 μL Fixed Volume Microdispenser, Drummond Scientific, Broomall, PA, USA) to dispense the gel solution (Figure 2b). The disposable syringe was held in a metal fixture with coolant solution inside to prevent gelation of the neutral collagen solution, as described in our previous study [11]. A 27G needle (NN-2719S, TERUMO, Tokyo, Japan) was connected to the tip of the syringe to microspot the gel solutions. The microinjector was inclined 18° to level the tapering of the connected needle. The solution in the syringe was injected at the rate of 2 μL/s using a motorized stage, or the solution in the microdispenser was injected manually. The position of the spotting stage was controlled using three motorized stages in three axial directions. The horizontal position was controlled using two motorized stages (TSDM60-20X, Opto-Sigma, Japan), and the vertical position was controlled using a motorized stage (SGSP26-50(X), Opto-Sigma, Japan). The rate of stage movement was set at 2.45 mm/s. All four motorized stages for the injector and stage position control system were connected to a personal computer using a commercial control software (LabVIEW 2015, National Instruments, Austin, TX, USA). 

Firstly, the spotting stage was elevated under the needle connected to the syringe, and the collagen or alginate solution was injected. After the injection, the stage was moved down in the direction of z-axis and moved horizontally to the next spotting position. By repeating this process, the spotting device enables arraying of the gel microconstructs containing cells (Figure 2c and Supplementary Material Video S1), and the spot diameter and cell number contained in the gel construct can be controlled uniformly by our spotting device.

### 2.3. Experimental Analyses of Diffusion Process of Cytokines in Core Layer of Gel Construct

In this study, we propose a gel microconstruct culture with three layers to simulate the development of skin and regenerate skin structures with appendage tissues (Figure 1). The diffusion of FGF-2 from the core layer was experimentally analyzed using a fluorometric method. 

**Figure 3:**
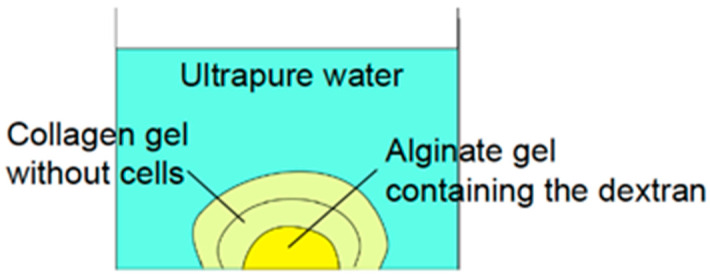
Experimental evaluation of diffusion phenomena of cytokines contained in core layer of gel construct. Diffusion of cytokines was simulated by the diffusion of FITC-dextran.

As shown in Figure 3, fluorescein isothiocyanate (FITC)-dextran (average mol wt = 4000) was used as the simulant for FGF-2. First, 1 μL of 1% FITC-dextran in 2% sodium alginate solution was spotted onto a ϕ35 Petri dish to form the core layer of microconstruct. Then, 5 mL of 2% CaCl_2_ solution with 1% FITC-dextran was placed on a Petri dish for gelation of alginate droplets. For complete gelation, a dish containing alginate gel spots was incubated overnight. Thereafter, the supernatant was aspirated, and 2.4 mg/mL neutralized type I collagen solution was spotted on the alginate gel spots to form the middle and outer gel layers. The middle and outer layers were constructed using 3 μL and 5 μL of collagen solution, respectively. The collagen layers on the alginate spots were gelled by incubating in a CO_2_ incubator for 30 min. 

PBS (1.5 mL) was poured into a Petri dish containing 20 spots of gel constructs and incubated in a CO_2_ incubator. To observe the diffusion process of FITC-dextran, 10 μL of the supernatant was collected from 1.5 mm above the gel spots, and the intensity of green fluorescence was measured using a biophotometer (Qubit 2.0 Fluorometer, Life Technologies, Carlsbad, CA, USA) at 0, 2,4, 6, 8, and 12 after the start of incubation. The intensity was normalized to that measured after 12 h of incubation. 

### 2.4. In Vitro Culture of Cell-Laden Three-Layered Gel Constructs

Mouse fetal epidermis-derived cells were isolated from fetus in female C57BL/SLC mice at GD17 [12]. First, fetuses were surgically excised from the mice under general anesthesia. Fetal epidermal tissues were harvested using a scalpel or surgical scissors and disinfected with povidone-iodine. Thereafter, the excised tissues were treated with 0.25% trypsin at 4 °C for 16 h. The digested tissues were cultured and gently agitated for 40 min in DMEM with 10% FBS, 100 IU/mL penicillin, 100 IU/mL kanamycin, 10 ng/mL cholera toxin, 10 ng/mL EGF, 0.5 μg/mL hydrocortisone, 5 μg/mL insulin, and 2 × 10^−9^ M triiodothyronine. Tissue debris was removed from the skin-digested solution using a 70 μm nylon cell strainer (Becton Dickinson, Franklin Lakes, NJ, USA). Finally, fetal epidermis-derived cells were isolated by centrifugation at 1200 rpm for 5 min. All experiments were approved by the ethical committees at Keio University and St. Marianna University School of Medicine (No. 1505, 1548).

Mouse ESCs derived from the 129/Ola strain (EB3 cell line, Riken Bioresource Center, Tokyo, Japan) were used to fabricate the second layer of microconstructs. EB3 cells were thawed from cryovials and expanded for 3 days on gelatin-coated Petri dishes in Glasgow Modified Essential Medium (GMEM) with 10% FBS, 1% antibiotic-antimycotic, 0.1 mM nonessential amino acid, 0.1 mM 2-mercaptoethanol, 0.1 mM sodium pyruvate, 10 μg/mL blasticidin S, and 1000 U/mL leukemia inhibitory factor. The cell cultures were maintained in a humidified tissue culture incubator at 37 °C and 5% CO_2_. After cell expansion, mESCs were dissociated and collected using 0.25% trypsin for gel microconstruct fabrication.

The three-layered gel microconstructs (Figure 1) were fabricated using the precise gel-spotting device developed in this study. Briefly, 1 μL of 400 ng/mL FGF-2 containing 2% alginate solution was dispensed onto a ϕ60 Petri dish to generate 16 alginate-gel spots containing FGF-2. The alginate gel droplets were incubated and gelled overnight at 4 °C in 5 mL of 2% CaCl_2_ solution containing 400 ng/mL FGF-2. After the complete gelation of alginate droplets, the CaCl_2_ solution was aspirated. Next, the alginate gel layer was covered with 3 μL of type I neutralized collagen solution containing mESCs and allowed to gel for 20 min in a CO_2_ incubator. Finally, 5 μL of type I neutralized collagen solution containing mouse fetal epidermal cells was dispensed to cover the collagen gel layer containing mESCs and allowed to gel for 20 min in a CO_2_ incubator. Finally, the gel microconstructs were washed 2 times and covered with a cell culture medium. The collagen gel culture method has been described previously [11,13,14]. Each three-layered gel construct contained 1.6 × 10^4^ mESCs and 1.6 × 10^4^ mouse epidermal cells. To evaluate the effects of cellular organization and FGF-2 on the reconstruction of hair follicle-like structures, three-layered constructs without FGF-2 in the core layer, and constructs with mESCs and epidermal cells in a single layer were fabricated and cultured under the same conditions (Table 1).

The gel microconstructs were cultured in papilla cell growth medium (PCGM, Toyobo, Japan) + 1% FBS + 0.5% Insulin Transferrin Triiodothyronine (ITT, Toyobo, Japan) + 1% Bovine Pituitary Extract (BPE, Toyobo, Japan) + 0.5% cyproterone acetate (Cyp, Toyobo, Japan) for the first 7 days to promote cell differentiation. Following the first 7-day culture, the constructs were cultured in GMEM with 10% FBS and 1% antibiotic-antimycotic to promote tissue reconstruction. The culture medium was exchanged every two or three days. The cell-laden gel constructs were cultured for 28 days. 

### 2.5. Histological Analysis

To detect the reconstruction of hair follicles and the surrounding structures, cell-laden gel constructs were processed for histological analysis. The gel beads were washed with 5 mL PBS three times and fixed with 10% formalin, processed for standard paraffin embedding, and sectioned into pieces of 3–4 μm thickness. Slides were immersed in xylene three times for deparaffinization, soaked in 100% ethanol twice for 5 min, 90% EtOH, 80% EtOH, and 70% EtOH each for 5 min, and deionized H_2_O for 1 min to perform rehydration. 

After rehydration, slides were stained with hematoxylin and eosin and observed under a microscope (BX51, Olympus, Tokyo, Japan). Hematoxylin and eosin staining was performed on constructs cultured for 28 days. 

The paraffin sections were deparaffinized and rehydrated for immunohistochemistry. Antigen retrieval was performed by boiling the slides in 10 mM citrate buffer (pH 6.0) for 10 min. Slide were cooled and immersed in PBS(-)three times. Nonspecific binding was blocked by incubation with an immunoblock solution (Protein Block Serum-Free Ready-to-Use, Dako, Santa Clara, CA, USA) for 30 min. Tissue sections were incubated with primary antibodies at room temperature for 60 min and washed with PBS. The following antibodies were used: monoclonal mouse anti-human smooth muscle actin (Dako, USA) and monoclonal mouse anti-human cytokeratin AE1 + AE3 (Dako, USA). The sections were then incubated with secondary antibodies (Envision+ system-HRP-labeled polymer anti-mouse, Daoko, USA) for 30 min. The color development was performed using DAB solution for 30–60 s. Nuclei were counterstained with hematoxylin. Stained tissue sections were observed under a microscope (BX51, Olympus, Japan).

## 3. Results

### 3.1. Diffusion Process of Cytokines in Core Layer of Gel Construct

**Figure 4:**
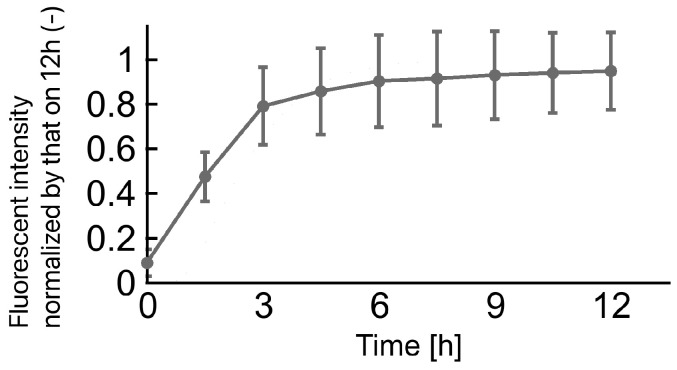
Time course of FITC-dextran concentration released from core layer of three-layered bead. Mean ± S.D., n = 5.

The fluorescence intensity of FITC-dextran in the supernatant of the gel constructs increased within 12 h of incubation (Figure 4). This result indicates that FITC-dextran was released from the core layer of gel construct within 12 h, and the concentration of dextran reached a plateau after 12 h of incubation. In particular, the amount of FITC-dextran in the supernatant increased during the first eight hours from the start point of incubation. Therefore, diffusion of FITC-dextran occurred mainly during the first eight hours. 

### 3.2. In Vitro Reconstruction of Hair Follicle-like Structure

**Figure 5:**
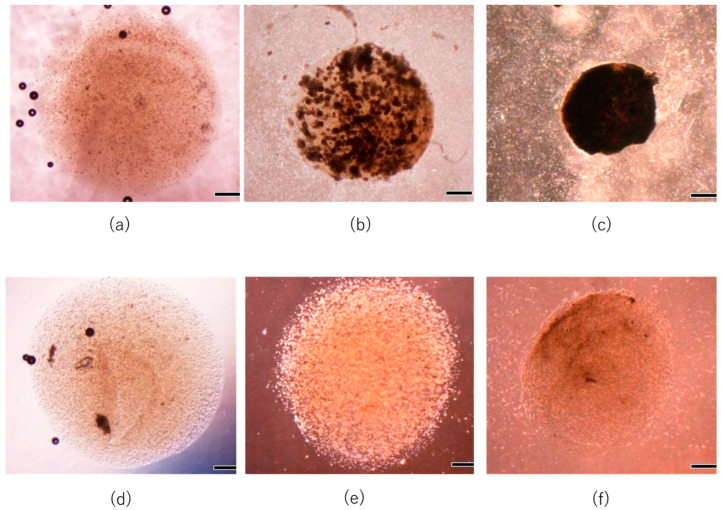
Representative images of cultured constructs in FGF(+)-layered group at day 0 (**a**), 7 (**b**), and 14 (**c**) and in FGF(−)-layered groups at day 0 (**d**), 7 (**e**), and 14 (**f**). Scale bar: 500 μm.

Representative microscopic images of the cultured specimens with and without FGF-2 are shown in Figure 5. Cells cultured with FGF-2 formed aggregate-like structures on day 7, and the specimen became opaque and contracted. This result indicates that the cells cultured with FGF-2 generated a dense extracellular matrix with tissue shrinkage. On the other hand, cells cultured without FGF-2 did not form aggregates and promoted slight tissue for 14-day culture. 

**Figure 6:**
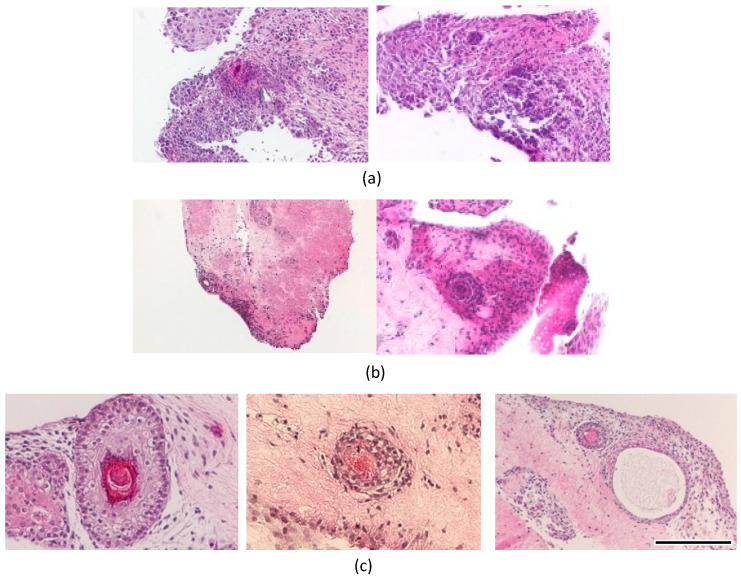
Hematoxylin–eosin stain of beads cultured for 28 days: (**a**) FGF(+)-mixed, (**b**) FGF(−)-layered, and (**c**) FGF(+)-layered. Scale bar: 200 μm.

**Figure 7:**
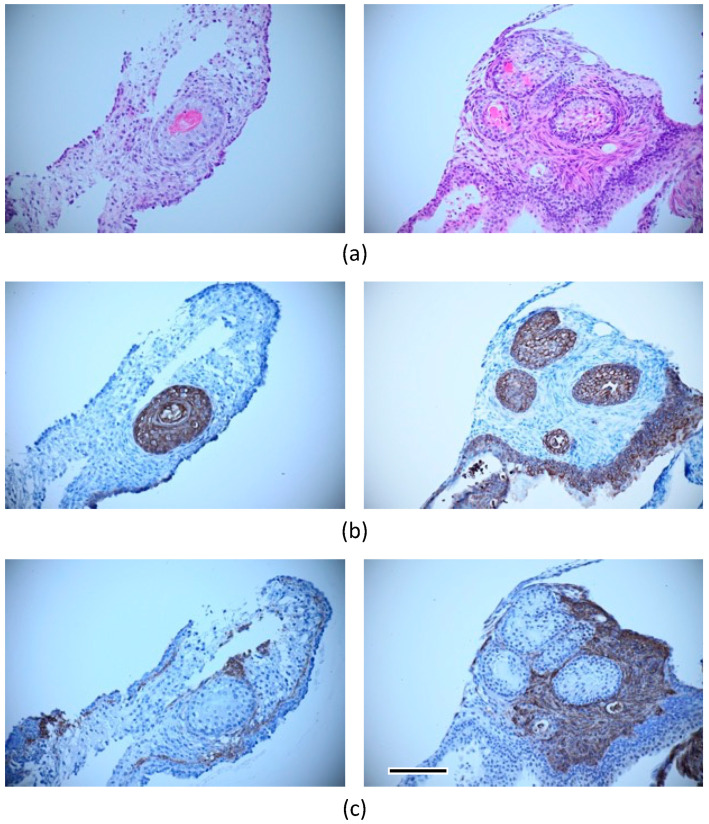
Immunostaining of cultured beads under hair follicle differentiation (FGF(+)-layered group): (**a**) hematoxylin–eosin, (**b**) cytokeratin, and (**c**) smooth muscle actin staining. Scale bar: 100 μm.

**Figure 8:**
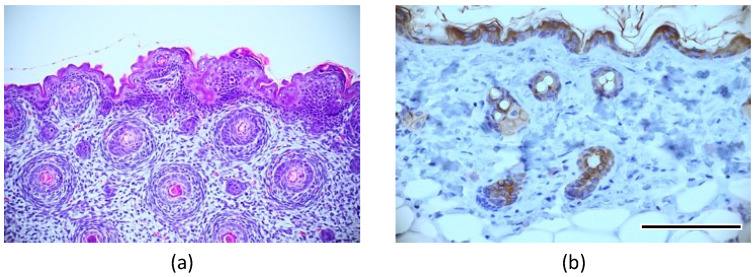
Immunostaining of “native” structure of fetal mouse skin using (**a**) hematoxylin–eosin and (**b**) cytokeratin. Scale bar: 200 μm.

In the mixed culture of epidermal and ES cells with FGF-2, the collagen gel disappeared, and cells proliferated with partial necrosis in the gel constructs cultured for 28 days (Figure 6a). The differentiation of cells in these specimens could not be determined from the tissue sections stained with hematoxylin and eosin. In the double-layered culture of epidermal and ES cells without FGF-2, a layered tissue-like epithelial structure was reconstructed in the superficial zone of gel constructs. In particular, a concentric structure with a keratinized center stained with eosin was observed in only one of the twenty specimens in the experimental group (Figure 6b). In the triple-layered culture of epidermal and ES cells with FGF-2 containing gel, concentric structures with keratinized center were frequently observed. Each concentric structure had a bilayered tissue of flat epithelial cells and a central structure of keratinized tissue (Figure 6c). Concentric structures were observed in nine of the twenty specimens. A sweat gland-like luminal structure near the concentric structure was also observed in the experimental group (right image in Figure 6c). Immunohistochemical analysis was performed to elucidate the tissue configuration. Concentric structures were positively stained with cytokeratin AE1+AE3, and adjacent tissues were stained with smooth muscle actin (Figure 7). The same histological analyses were performed on the dorsal skin of mice. Similar concentric structures with keratinized center and a bilayer of flat epithelial cells were observed in the gel constructs cultured *in vitro*, as compared to those in the “native” skin of mouse (Figure 8). 

## 4. Discussion

Here, we developed a microgel-spotting device and proposed a multilayered gel culture to simulate the early developmental stages of skin appendages. During the early development of follicles, different cells are organized to form multilayered structure resembling a “telescope” [15]. Moreover, a cytokine concentration gradient is generated during follicular development. Our culture method simulated this tissue organization at an early stage to form a three-layered gel structure containing FGF-2 in an agarose gel as the core layer, mouse ESCs in a collagen gel as the middle layer, and fetal mouse epidermal cells in a collagen gel layer as the outer layer. 

A gel solution-spotting device was developed to fabricate a size-controlled array of gel constructs. Triple-layered gel constructs were fabricated, and the generation of a cytokine gradient was confirmed using FITC-dextran diffusion experiments. Based on the results of the diffusion experiments, the concentration gradient was maintained for approximately 12 h. In this study, the formation of hair follicle-like constructs was observed in a multilayered culture of ESCs and fetal epidermal skin cells with a concentration gradient of FGF-2. These follicle-like constructs observed in the gel constructs resembled “native” hair follicles in mice skins from the results of histological analysis. Therefore, the maintenance of the FGF-2 concentration gradient for the first 12 h of culture was considered sufficient to stimulate hair follicle differentiation. The requirements for cytokines and other chemical factors have been reported in previous studies [1,2,10,16]. In contrast, hair follicle-like constructs were not observed in mixed cultures of ESCs and epidermal skin cells with an FGF-2 concentration gradient. This indicates the necessity of cellular organization for the *in vitro* regeneration of hair follicles as described in the previous study [15]. Frequency of hair follicle development was 45% (9/20 specimens) in layered culture of ESCs and epidermal cells with an FGF-2 gradient, whereas it was extremely low (1/20 specimens) in the cultures without an FGF-2 gradient. Therefore, we speculated that cellular organization and FGF-2 gradient are critical factors for *in vitro* hair follicle regeneration. Moreover, adjacent tissues around the hair follicle-like constructs stained positively for anti-smooth muscle actin and were generated under a multilayered culture with an FGF-2 gradient. Lindner reported the effect of FGF-2 dose on the proliferation of smooth muscle cells [17]. Reconstruction of smooth muscle tissue may have been stimulated by the FGF-2 gradient in our culture model. As compared to the immunohistochemistry of “native” mouse skin, these muscle tissues could be considered as arrector pili muscle. To pursuit the precise and comprehensive identification of hair follicle structures, specific markers such as CD49f, Krt10, Loricrin, Krt15, and Lhx2 should be evaluated.

In this study, the regeneration of hair follicle-like structures was observed in the culture 21–28 days after the fabrication of multilayered gel constructs. The development of “native” mouse hair follicles begins around GD17–18 when the epidermal cells begin to thicken and form placodes. Hair follicles are generated after the formation of placodes, and they mature around GD21 [18,19]. Considering the time course of “native” hair follicle development, our culture model can simulate the environment of *in vivo* hair follicle development. Our multilayered gel culture model has the potential to reconstruct epidermis, arrector pili muscle, sweat gland-like luminal tissue, and hair follicle-like structures. However, our culture method did not regenerate hair roots and shafts *in vitro* for longer culture periods. As described in Figure 1, the thickness of the second and third layers was not homogeneous in our multicellular culture model. This inhomogeneity of thickness might affect the interaction of mESCs and fetus-derived mouse epidermal cells to promote the reconstruction of follicle-like structures. Optimization of culture conditions and additional factors is required for complete *in vitro* hair regeneration. 

## 5. Conclusions

In this study, we developed a multilayered gel-spotting device and proposed a three-dimensional culture method to simulate the environment of hair follicle development during *in vitro* hair follicle regeneration. A mechanical microgel-spotting device was developed to construct a multilayered gel culture containing mouse ESCs and fetal epidermal cells with a cytokine gradient. Hair follicle-like constructs with appendages (epidermis, sweat glands, and arrector pili muscles) were formed in a multilayered culture with a cytokine gradient. 

## Figures and Tables

**Table 1:** Different experimental groups used to evaluate the effects of cellular organization and cytokine concentration gradient.

Experimental Group	FGF-2	Gel Spot Structure (2nd and 3rd Layer)	Evaluation	
FGF(+)-mixed	(+)	Mixed	Cellular organization	
FGF(+)-layered		Layered		Cytokine concentration gradient
FGF(−)-layered	(−)			

## Data Availability

The data from this work will be shared and made available upon request to the corresponding author.

## Supplementary Materials

The following supporting information can be downloaded at https://www.mdpi.com/article/10.3390/mi14091651/s1. Video S1: Movement of the precise gel-spotting device constructed by linear motorized stages.
